# The Impact of Social Media on Body Image Perception in Young People

**DOI:** 10.3390/nu17091455

**Published:** 2025-04-26

**Authors:** Natalia Czubaj, Martyna Szymańska, Beata Nowak, Mateusz Grajek

**Affiliations:** Department of Public Health, Faculty of Public Health in Bytom, Medical University of Silesia in Katowice, 41902 Bytom, Poland; gmattgrayek@gmail.com (N.C.); s82642@365.sum.edu.pl (M.S.); s82615@365.sum.edu.pl (B.N.)

**Keywords:** social media, self concept, body image

## Abstract

Background: Social media can significantly impact body image perception among adolescents. Objective: This study examines how exposure to fitspiration content relates to body esteem, with a focus on gender differences and BMI. Methods: A cross-sectional online survey was conducted among 211 participants using validated instruments (Body Esteem Scale, A. Sobczak’s silhouette scale). Data were analyzed using descriptive statistics and chi-square tests. Results: Exposure to athletic images decreased self-esteem in 37% of participants, particularly among women (*p* = 0.004). Gender significantly influenced the tendency to compare oneself with athletic individuals (*p* = 0.02). BMI was not significantly associated with body image perception (*p* = 0.05). Conclusions: Gender-specific patterns in social media-related body image perception highlight the need for targeted interventions to mitigate negative impacts among young adults.

## 1. Introduction

Social media (SM) shape the standards of what is considered desirable and attractive, resulting in the creation of an unrealistic and difficult-to-attain image of the ideal body. SM users are exposed to a distorted representation of reality that does not reflect the actual state of affairs. The pursuit of this ideal can contribute to mental health issues. Available research indicates a significant correlation between eating disorders, depression, and the aspiration to achieve the body image portrayed on social media [1,2]. However, not every user is susceptible to negative influences. This depends on individual behaviors, habits, and sensitivity to external factors. Self-assessment of one’s body is often based on comparisons with peers. However, in today’s world, these comparisons increasingly include the idealized body images presented on social media [3].

In the past, the image of the ideal body was presented through mass media, such as television and magazines, regardless of audience interest. Today, SM users have the autonomy to choose the content they consume. The term “social media” refers to mobile applications or websites that allow users to create and share content, communicate instantly, express themselves, and engage in virtual communities. However, SM use can have negative consequences, including dissatisfaction with one’s appearance and the adoption of unhealthy eating habits in pursuit of the socially accepted ideal body shape [3]. Self-esteem is often shaped by comparisons with peers, with a tendency to compare ourselves more frequently to those perceived as more attractive. However, such assessments are subjective, exaggerated, and often misaligned with reality. In the context of social media, there is an additional risk of comparing oneself to users we have never met in real life, which can lead to a distorted perception of self-worth [4].

SM can be leveraged to promote healthy eating habits among young people, given their high level of engagement with these platforms. Presenting pro-health behaviors on SM may contribute to improved dietary choices [5]. However, food manufacturers take advantage of SM influence on young adults and children by advertising their products without considering their nutritional value. Moreover, much of the information shared on SM does not stem from scientific research, which can lead to confusion among users who lack the necessary skills to critically assess the accuracy of available information [6,7]. Manufacturers of supplements and other nutritional products may prioritize potential profits over the health impact on consumers in their marketing strategies [8]. Additionally, marketing specialists collaborate with well-known SM personalities to enhance trust in the advertised products. This strategy relies on leveraging the audience’s trust in the content creator [9].

The relationship between SM and its users is influenced by the pervasive presence of online hate. Many users feel emboldened to publicly insult others without consequences due to the anonymity these platforms provide. Research suggests that one does not need to be a direct target of online hate—merely witnessing it can negatively impact subjective well-being [10]. Online hate is a highly complex phenomenon, yet it poses a significant risk to mental health, particularly among young individuals [11].

SM users have full control over what aspects of their lives they choose to share. This selective presentation of content can be problematic for audiences who, when comparing themselves to others, only see carefully curated and idealized moments. Particularly concerning is the tendency to compare one’s own appearance to the highly polished images presented on SM, which can negatively impact body perception. Research suggests that the influence of social media on self-esteem is rooted in social comparison theory, with this relationship being indirectly shaped by prevailing beauty standards [12]. Narcissism, as a multidimensional personality trait, is prevalent among SM users. Gaining likes and positive comments allows narcissistic individuals to attract attention, reinforce their self-worth, and craft an idealized portrayal of their lives through carefully curated content [13]. Audiences consuming such content are particularly vulnerable to making comparisons, often perceiving their own appearance and quality of life as inferior [14].

The promotion of a healthy lifestyle is highly popular on SM, however, the risk of developing eating disorders among young individuals remains significant. Each user chooses whom to follow, yet even influencers who serve as role models may struggle with various disorders themselves [15]. The beauty industry also plays a role in shaping perceptions of physical appearance. Many brands suggest to their target audience that certain products are essential tools for achieving an ideal look [16]. Comparing oneself to others can further distorted body perception, increasing the likelihood of unhealthy eating behaviors and eating disorders [17].

Eating disorders (ED) are defined as abnormal eating behaviors accompanied by an excessive preoccupation with body weight, negatively impacting physical health or psychosocial functioning. EDs are serious psychiatric illnesses associated with high morbidity and mortality rates. The primary eating disorders include Anorexia Nervosa, Bulimia Nervosa, Orthorexia Nervosa, Binge Eating Disorder (BED), and Bigorexia Nervosa [18].

Anorexia Nervosa (AN) is an eating disorder characterized by a severe restriction of calorie intake, leading to significantly reduced body weight. Common behaviors associated with AN include calorie counting, excessive portion control, compulsive exercise, and purging methods such as self-induced vomiting or the use of diuretics. Individuals with AN experience an intense fear of weight gain and have a distorted perception of their body image [19]. AN is more prevalent among women than men. Risk factors for its development include childhood overweight, mood fluctuations, personality traits such as impulsivity and perfectionism, a history of sexual abuse, and concerns about body weight influenced by family or peers [19,20].

Bulimia Nervosa (BN) is an eating disorder characterized by episodes of extreme overeating, followed by compensatory behaviors aimed at preventing weight gain. Bulimia is more common in women than in men and has a higher prevalence than Anorexia Nervosa. Individuals with bulimia consume unusually large quantities of food—significantly more than a healthy person would eat in similar circumstances and within a comparable timeframe. During binge-eating episodes, they experience a loss of control over food intake [21]. Compensatory behaviors associated with bulimia include self-induced vomiting, severe calorie restriction, the use of laxatives or diuretics, enemas, and the misuse of medications such as thyroid hormones or insulin without medical supervision [22].

Orthorexia Nervosa (ON) is defined as an obsessive focus on consuming foods perceived as healthy. Individuals with ON follow strict dietary rules, eliminate entire food groups from their diet, and excessively focus on the origin, preparation, and nutritional value of their food. The condition is characterized by feelings of anxiety and distress, as well as disruptions to daily functioning due to rigid dietary restrictions [23]. Risk factors for ON include personality traits such as perfectionism, a tendency toward obsessive-compulsive behaviors, dissatisfaction with one’s appearance, a history of dieting, and exposure to idealized body images promoted in the media [24,25].

Binge Eating Disorder (BED) is the most common eating disorder, characterized by episodes of uncontrollable hunger, during which large quantities of food are consumed in a short period. During these episodes, the amount of food intake is significantly higher than what would be expected in similar circumstances. A key distinguishing feature of BED is the absence of compensatory behaviors, such as excessive physical activity or self-induced vomiting [18,26]. Additionally, BED is associated with specific eating behaviors [27]:

- Meals are consumed more quickly than by healthy individuals.
- Eating continues until an uncomfortably full sensation is reached.
- Large amounts of food are consumed despite the absence of physical hunger.
- Meals are often eaten in solitude due to shame over the quantity of food consumed.
- After a binge episode, individuals with BED may experience self-disgust, depression, or intense guilt.

Risk factors for developing BED include: substance abuse, polymorphisms of DRD2, OPRM1, 5-HTT or MC4R, alterations in the composition of the gut microbiota, abnormalities in the connectivity of the cortex, character traits i.e., perfectionism, childhood obesity, family conflicts, weight pressures from family, physical or sexual abuse, loss of control over food intake in childhood [26].

Bigorexia Nervosa, also known as muscle dysmorphia, is a disorder characterized by an obsessive pursuit of increased muscle mass while simultaneously reducing body fat. Individuals with bigorexia often adopt unhealthy dietary habits and engage in excessive physical activity to achieve their desired appearance. Negative body perception and appearance-related anxiety are particularly common among young people, potentially leading to severe health and psychological consequences. Additionally, improper eating habits that contribute to eating disorders may also play a role in the development of bigorexia [28,29]. Other risk factors for bigorexia include an unhealthy relationship with food, peer pressure, media influence, and personality traits such as perfectionism [29,30]. Studies have shown a connection between the obsessive pursuit of muscle gain, bigorexia, and disordered eating behaviors [31].

Social media (SM) have a significant impact on users’ behaviors and perception of reality. While they can be used positively to promote a healthy lifestyle without excessive restrictions, they may also increase the risk of developing various eating disorders (EDs). A particularly dangerous aspect is the tendency to compare oneself to online celebrities and adopt extreme dietary methods in an attempt to achieve a similar appearance [32]. A well-balanced diet, along with regular physical activity, are essential for overall health. However, in the context of EDs, diet and exercise can become an obsession. Disorders such as Anorexia Nervosa, Bulimia Nervosa, Orthorexia Nervosa, Bigorexia, and Binge Eating Disorder pose a significant risk to social media users [33].

One of the key theoretical foundations for understanding the influence of social media on body image is social comparison theory, which posits that individuals determine their own social and personal worth based on how they stack up against others. In the context of social media, constant exposure to idealized images intensifies upward comparisons, often leading to dissatisfaction with one’s own body and life. Despite extensive research linking social media usage to body image disturbances, few studies have comprehensively addressed how gender-specific mechanisms influence this process among young adults.

Research questions:

(1) How does the exposure to fitspiration content on social media affect body image perception among young adults?
(2) Are there gender differences in the tendency to compare oneself with athletic individuals online?
(3) What is the role of BMI in moderating body image perception in the context of social media exposure?

## 2. Material and Methods

The study was conducted using a questionnaire consisting of seventeen original questions examining the impact of social media on body image perception, the Body Esteem Scale (BES), and A. Sobczak’s silhouette scale representing different levels of nutrition. The questions were in a closed-ended, single- and multiple-choice format. The study sample included 211 respondents. The inclusion criteria required participants to be at least 18 years old and active social media users. Participants were recruited via Facebook and Instagram, which, while allowing broad reach, introduces potential selection bias by favoring individuals who are more engaged with these platforms.

The questionnaire was distributed online via Facebook and Instagram. Respondents were asked about the types of social media they use, and the survey covered topics such as social comparison, dietary authority, and the influence of social media on behaviors and perceptions of reality. Silhouette scales were used to assess participants’ self-esteem. The survey was conducted electronically via Google Forms between November 2022 and March 2023.

The Body Esteem Scale (BES) used in this study has been previously validated for young adult populations and demonstrates high internal consistency (Cronbach’s alpha > 0.85). Similarly, A. Sobczak’s silhouette scale has been validated in Polish samples and is commonly used to assess self-perceived body shape.

Formal ethical approval was not required for this study due to its anonymous and non-invasive survey-based nature, in accordance with national and institutional guidelines. Nevertheless, the research was conducted in full compliance with the principles of the Declaration of Helsinki (2013 revision). All participants were informed about the aim of the study, the voluntary nature of participation, and their right to withdraw at any time without any consequences. No personally identifiable information was collected. Data confidentiality and anonymity were strictly maintained throughout the study. Participants provided informed consent electronically before completing the survey.

The obtained results were analyzed using Statistica for Windows 13.3 with descriptive (n, percentage) and comparative statistics (Chi-square test). A significance level of *p* < 0.05 was considered statistically significant. Missing data were handled using listwise deletion. Chi-square tests were applied to assess associations between categorical variables, with significance set at *p* < 0.05.

## 3. Results

The study included 211 respondents, with an average age of 27 ± 0.01 years. The sample consisted of 69 men (32.7%) and 142 women (67.3%). Regarding education levels, 49.8% (N = 105) of respondents had secondary education, 37% (N = 78) held higher education degrees, 7.6% (N = 16) had vocational education, while 2.8% (N = 6) had completed middle school, and another 2.8% (N = 6) had primary education.

Associations between demographic variables and social media usage patterns presented in Table 1, Table 2, Table 3, Table 4, Table 5 and Table 6 were tested using chi-square tests where applicable. Only significant results are reported.

Data was collected on the choice of social media platforms, the most frequently used ones, and the thematic preferences of the content viewed. Participants’ activity levels on social media were also considered. The collected data are presented in Table 1.

Respondents spend the most time on Instagram—143 (67.8%) of the surveyed individuals. Among the most common interests of respondents were content related to healthy eating (54.5%, N = 115), travel (51.7%, N = 109), and sports & gym (47.4%, N = 100). The vast majority of respondents are active on social media and share content on public profiles—95 (45%) of the participants.

Next, respondents’ feelings about the content published on social media were analyzed, along with their opinions on photo enhancement and the use of retouching features. Table 2 presents the collected results.

The opinions of the respondents regarding the use of beauty filters were divided—72 (34.1%) respondents admitted to occasionally using beauty filters and did not have a negative opinion about them. In contrast, 71 (33.6%) respondents stated that using such filters distorts reality. The photo retouching feature is occasionally used by 33.6% (N = 81) of respondents. Content shared on social media by physically fit individuals most often leads to a decrease in self-esteem for 37% (N = 78) of respondents. More than half of the respondents (N = 114) reported the existence of several user accounts that impress with their appearance.

The respondents’ trust in content published by other social media users was assessed. The data is presented in Table 3.

Nearly 48% (N = 101) of respondents believe that knowledge of healthy eating is not directly related to having a fit physique. A significant majority, 82.9% of respondents, believe that the number of followers is not an indicator of a user’s high value. Over half of the respondents (N = 108) sometimes follow product recommendations made by celebrities. In the context of promoting misleading information on social media, 31.3% (N = 66) of participants agreed with the statement that celebrities should not comment on the principles of healthy eating.

The respondents’ attitudes toward content shared on social media by other users were examined. The data is presented in Table 4.

Only 27.5% (N = 58) of respondents find inspiration in the dietary habits presented on social media by nutrition experts. A significant majority—46.9% (N = 99)—sometimes compare themselves to famous, fit individuals on social media in terms of physical appearance. The most common motivation for taking care of oneself and one’s physique among respondents is achieving a better appearance, cited by 140 (66.4%) participants. For 45% (N = 95) of respondents, their perception of their own body has changed only slightly. Only 19% (N = 40) of respondents reported never feeling that their life is less attractive compared to others on social media.

Table 5 presents respondents’ self-assessment regarding various body-related aspects.

Among all thirty-four criteria, the rating “I have strongly negative feelings” appeared 436 times (6%), the rating “I have moderately negative feelings” appeared 1446 times (20.1%), the rating “I have no feelings” appeared 2226 times (31.1%), the rating “I have moderately positive feelings” appeared 1936 times (27%), and the rating “I have strongly positive feelings” appeared 1130 times (15.8%).

**Figure 1:**
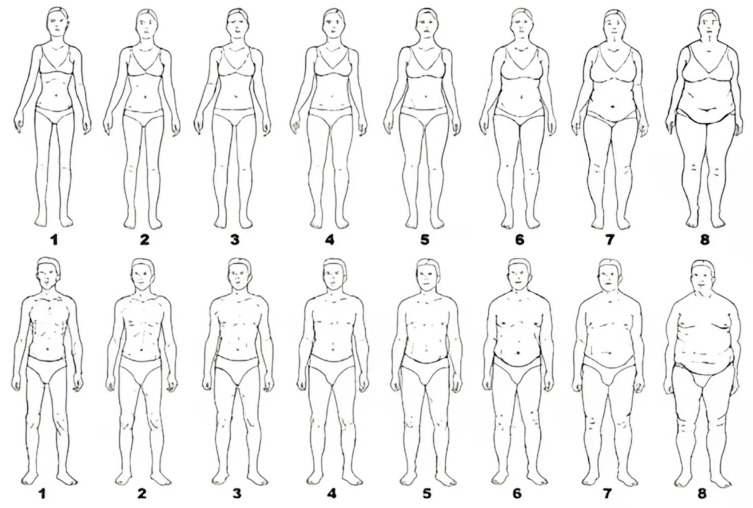
Body shape scales with different nutritional statuses according to A. Sobczak.

Based on Figure 1, respondents were asked to select and mark the body type that most closely resembles their physique. The respondents’ choices are presented in Table 6.

Respondents most frequently rated their body type with the numbers: 4 (52 people, 24.8%), 5 (49 people, 23.3%), 3 (42 people, 20%), and 8 (40 people, 19%).

An analysis of the relationship between gender and selected parameters was conducted. The influence of gender on the perception of athletic individuals in social media (SM) was examined. The collected data is presented in Table 7.

In addition to statistical significance, effect sizes were calculated (Cramer’s V for Chi-square tests). The association between gender and perception of athletic individuals on social media showed a small-to-moderate effect size (Cramer’s V = 0.22), suggesting that while gender differences are statistically significant, they are of modest practical importance.

A statistically significant relationship was found between gender and the way respondents perceive photos of athletic individuals (*p* = 0.004) as well as comparing themselves to athletic individuals on social media (*p* = 0.02).

The relationship between respondents’ BMI levels and their perception of their own body and life was analyzed. The results of this analysis are presented in Table 8.

No statistically significant relationship was found between BMI and the perception of one’s own body as a social media user (*p* = 0.05) or the evaluation of one’s own life (*p* = 0.9).

## 4. Discussion

The main findings of this study indicate that gender significantly affects how young adults perceive fitspiration content on social media, with women showing greater self-criticism. Furthermore, BMI did not significantly impact self-perception outcomes. These findings provide new insights into the gendered dynamics of social media influence on body image.

Social media has become an inseparable part of many people’s lives. They serve as a form of entertainment, a means of work and income, and a platform for exchanging views, opinions, and sharing life experiences. Just as in their early days, they primarily fulfill a communicative function, enabling users to create or strengthen relationships even over long distances. However, despite the seemingly easier access to social interactions, numerous studies report that social media users often experience greater loneliness and lower well-being compared to those who use these platforms more moderately [34,35,36].

Feelings of loneliness and decreased body satisfaction have been linked to a higher risk of eating disorders in the study by Pop, Iorga, and Iurcov [37]. The reported sense of loneliness was associated with more frequent use of social media, which, in turn, was positively correlated with an increased risk of eating disorders.

Many studies emphasize the significance of gender in the context of increased susceptibility to content presented on social media. A study conducted by Choukas-Bradley et al. indicated that girls are more vulnerable to the negative effects of social media use than boys [38]. Negative effects are understood to include lowered self-esteem, dissatisfaction with one’s appearance, and potential eating disorders. Females were found to engage in social comparison with other users and celebrities more frequently, which could impact their psychological well-being [38]. Regardless of gender, individuals with low self-esteem may compare their bodies to the appearance of well-known and athletic individuals. Boys are also exposed to the harmful effects of social media, as confirmed by research conducted by Vuong et al. [39]. The authors observed that the negative effects of social media use on boys differ from those on girls. While girls tend to focus on body slimness, boys show a greater interest in visible musculature. The desire for acceptance and attractiveness is characteristic of adolescence and typically applies to young people. Vall-Roqué, Andrés, and Saldaña conducted studies exploring the relationship between age and a distorted body image in connection with social media use [40]. The study was conducted without the participation of men, and women were divided into two age groups: 14–24 years and 25–35 years. The authors observed that the younger group of women was more susceptible to body dissatisfaction, low self-esteem, and the pursuit of a slimmer figure compared to the older group. A significant limitation of this study was the exclusion of the male group from the analysis.

The desire to be attractive and conform to current beauty standards is not limited to young people, although they are the most susceptible to online manipulation. Imperatori et al. found that an obsession with one’s appearance is strongly linked to the risk of developing eating disorders [41]. While physical activity is crucial for maintaining health, the authors emphasize that excessive physical activity driven by the desire to achieve a desired body weight among young individuals can be a dangerous phenomenon. The obsession with one’s appearance became a widespread issue during the COVID-19 pandemic when, due to restrictions, many people increased the time spent online and on SM. During this period, there was a noticeable increase in searches for terms related to eating disorders. The impact of the pandemic on social media use and body image was explored by Vaccaro et al. [42]. The authors emphasize the impact of social media on interpersonal relationships, with the content shared on users’ profiles that showcases physical appearance serving as a form of “first impression”. Nutley et al. also noted that during the COVID-19 pandemic, internet users sought psychological support through social media [43]. Scholars agree that the pandemic period and social isolation increased the frequency of social media use and heightened the risk of eating disorders, due to restrictions and difficulties in accessing medications and food, as well as fear of illness.

Currently, ED can take various forms. What once seemed like an increased appetite or an excessive focus on health and proper nutrition now raises suspicion of eating disorders. Canals and Arija Val, in their work, state that the excessive use of SM is positively correlated with a higher risk of developing eating disorders [44]. Furthermore, the researchers emphasize that social media can exacerbate existing eating disorders. Meanwhile, Dane and Bhatia observed that the choice of accounts followed depends on the individual user, with those more prone to eating disorders and lower self-esteem selecting accounts on social media that could worsen their condition. Moreover, users with a positive self-esteem tended to follow accounts that promoted self-acceptance [45]. In our study, the behaviors of respondents were analyzed in the context of their feelings after viewing images of athletic individuals. The participants declared that photos of athletic people on social media motivated 34.6% (N = 73) to take action, while 37% (N = 78) of the respondents stated that these images made them feel worse. The behaviors of the respondents and their feelings toward photos of athletic individuals may stem from the predispositions of the individuals themselves, that is, the average social media user. Future studies should also consider the temperament and characteristics of the individuals being studied, such as perfectionism, narcissism, or susceptibility to manipulation or addiction.

Despite numerous reports highlighting the negative impact of social media on users’ mental health, the beneficial effects of online resources should also be considered. The development of technology and the increased awareness of many active social media users also provide opportunities for educating other users. Research by He and Sun reports that social media does not necessarily have to negatively affect its audience. On the contrary, promoting sports participation and active leisure can have a positive influence on them [46]. Peter and Brosius, on the other hand, emphasize that social media can have a dual impact, and the choice of content lies with the user. Some social media users select content that may exacerbate their problems, while others may use social media to seek support and potential solutions for overcoming eating disorders [47]. Despite the desire to recover from eating disorders and explore supportive topics related to them, the average user may face challenges due to the algorithms operating on social media platforms. A study by Montag et al. presents findings on how algorithms work on social media. The goal of these platforms is to capture the user’s time. The longer photos, videos, or posts on a particular topic are viewed, the more the algorithm deems those topics attractive to the user, encouraging them to spend more time in the specific app [48]. As a result, the algorithm’s operation can hinder the search for health-promoting content within apps, thus obstructing the recovery process for individuals struggling with the issue.

The heightened self-criticism observed among women may be partially explained by internalization of societal beauty standards and higher levels of body surveillance, which are more prevalent among females according to objectification theory. Future interventions should address these gendered cognitive patterns by promoting critical media literacy and resilience against unrealistic beauty ideals.

Practically, mental health educators, school counselors, and social media platforms should collaborate to design programs that foster positive body image and reduce harmful social comparison processes, particularly among young female users.

This study has several limitations that should be considered. First, the cross-sectional design precludes causal inferences between social media use and body image perception. Second, the sample, recruited online via social media, may not be representative of the broader young adult population and may introduce selection bias. Third, self-reported measures are subject to social desirability bias and memory recall errors. Finally, the study did not control for psychological variables such as self-esteem, perfectionism, or narcissistic traits, which could moderate the observed relationships. Future research should incorporate longitudinal designs and a broader range of psychological covariates.

These findings underscore the urgent need for public health strategies that address the psychological effects of social media on young people’s body image. Prevention efforts should prioritize media literacy education, the promotion of body diversity, and the development of supportive online communities.

Future research should explore longitudinal changes in body image perceptions in relation to social media use, investigate protective factors such as resilience and critical thinking skills, and assess the efficacy of intervention programs designed to mitigate the negative impact of social media on mental health.

## 5. Conclusions

- Social media users are susceptible to imitating celebrities, and the majority of respondents reported feeling worse about their lives due to social media.
- The study demonstrated that gender influences how individuals perceive their bodies through the lens of social media, with the female group being more critical of themselves.
- BMI does not impact the way individuals perceive their bodies and lives.
- Our findings highlight the need for targeted public health interventions to address the negative effects of social media on body image perception, particularly among young women. Educational programs should focus on enhancing media literacy and promoting diverse and realistic body standards. Future longitudinal studies are recommended to explore causality and identify protective psychological traits.

## Figures and Tables

**Table 1:** Characteristics of the study group.

Question	Option	N (%)
Use of SM ^1^	Instagram	190 (90%)
	Facebook	186 (88.2%)
	TikTok	105 (49.8%)
	Snapchat	85 (40.3%)
Most frequently chosen SM	Instagram	143 (67.8%)
	Facebook	46 (21.8%)
	TikTok	14 (8.1%)
	Snapchat	5 (2.4%)
Preferred SM content topic ^1^	Healthy diet	115 (54.5%)
	Travel	109 (51.7%)
	Sports & gym	100 (47.4%)
	Animals	66 (31.3%)
	Parenting & childcare	59 (28%)
	Automotive	41 (19.4%)
	Celebrities	28 (13.3%)
Social media activity	Active (Publicly)	95 (45%)
	Active (Privately)	59 (28%)
	Passive	57 (27%)

^1^ Multiple choice question.

**Table 2:** Respondents’ perception of contents posted on SM.

Question	Option	N (%)
Opinion on the use of beauty filters	I consider it normal, I sometimes use them	72 (34.1%)
	I believe it distorts reality	71 (33.6%)
	I have no opinion, I don’t pay attention	63 (29.9%)
	I think it’s better to view aesthetically pleasing content, so using this feature is beneficial	5 (2.4%)
Use of photo retouching features	Sometimes	81 (38.4%)
	No	71 (33.6%)
	Yes	31 (14.7%)
	Passive social media use	28 (13.3%)
Perception of content from fit individuals	Lowered self-esteem	78 (37%)
	Motivation and inspiration to take care of oneself	73 (34.6%)
	Indifference	60 (28.4%)
Presence of users who impress with their appearance	There are a few	114 (54%)
	There are plenty of them	70 (33.2%)
	No such users	27 (12.8%)

**Table 3:** Trust in other SM users.

Question	Option	N (%)
Do you think that knowledge of healthy eating goes hand in hand with a fit physique?	Yes	82 (38.9%)
	No	101 (47.9%)
	No opinion	28 (13.3%)
Do you believe that the number of followers is an indicator of a user’s high value?	Yes	16 (7.6%)
	No	175 (82.9%)
	No opinion	20 (9.5%)
Do you follow product recommendations made by celebrities?	Yes	12 (5.7%)
	Sometimes	108 (51.2%)
	No	91 (43.1%)
Imagine your favorite “influencer” announces on their profile that they are going on a “starvation” diet and believe it is the most effective way to lose weight. What do you think about that?	Celebrities shouldn’t be commenting on healthy eating principles.	66 (31.3%)
	It’s just a passing trend to me, and they can do whatever they think is right.	62 (29.4%)
	I’m not sure what to think, because they are not a trained dietitian.	53 (25.1%)
	Since they are doing it, I’ll wait for the results and maybe I’ll do it too.	30 (14.2%)

**Table 4:** The respondents’ attitudes toward content shared on social media.

Question	Option	N (%)
Are the eating habits of celebrities an inspiration for you?	Yes	6 (2.8%)
	Sometimes	91 (43.1%)
	No	56 (26.5%)
	Only qualified dietitians	58 (27.5%)
Do you ever compare yourself (in terms of physical appearance) to famous, fit individuals on social media?	Yes	48 (22.7%)
	Sometimes	99 (46.9%)
	No	64 (30.3%)
What inspires you to take action and care for yourself and your physique? ^1^	Famous athletes and trainers	23 (10.9%)
	The desire to be better than your own friends	53 (25.1%)
	Taking care of your health from within	122 (57.8%)
	A better appearance for yourself	140 (66.4%)
Do you think your perception of your body has changed since becoming a social media user?	Yes	58 (27.5%)
	Only a little	95 (45%)
	Not at all	58 (27.5%)

^1^ Multiple choice question.

**Table 5:** Body self-assessment of respondents.

Body Aspects	I Have Strongly Negative Feelings	I Have Moderately Negative Feelings	I Have No Feelings	I Have Moderately Positive Feelings	I Have Strongly Positive Feelings
Body Odor	7	26	66	62	50
Appetite	10	44	50	62	45
Nose	8	54	61	51	37
Physical Endurance	17	48	78	51	17
Reflexes	1	29	60	77	40
Lips	11	32	58	66	44
Muscle Strength	10	50	79	53	19
Waist	10	54	68	52	27
Energy Level	17	59	64	56	15
Thighs	28	60	54	47	22
Ears	7	29	64	55	56
Arms	12	32	68	54	45
Chin	17	42	77	42	33
Body Build	15	59	61	56	20
Physical Coordination	9	46	74	56	26
Buttocks	11	43	58	63	36
Agility	11	29	71	62	38
Shoulders Width	13	35	67	62	34
Hands	11	26	69	71	34
Chest	12	59	62	57	21
Eves Appearance	7	30	46	56	72
Cheeks	8	37	75	55	36
Hips	11	40	72	55	33
Legs	18	47	64	55	27
Body Shape	16	49	60	64	22
Energy Level	10	24	64	61	52
Feet	19	43	72	49	28
Genitals	9	36	62	69	35
Stomach	22	64	71	33	21
Health	9	48	66	62	26
Sexual Activity	13	32	60	62	44
Body Hair	24	48	72	45	22
Face	10	40	67	61	33
Body Weight	19	52	66	54	20

Responded were asked “Below are various aspects related to your body. Please rate each aspect by selecting one of the five options”.

**Table 6:** Self-Assessment of Respondents Body Types.

Question	Option	N (%)
Look at the illustrations below. Try to match your body type to one of them (First row for women, second row for men)	1	8 (3.8%)
	2	24 (11.4%)
	3	42 (20%)
	4	52 (24.8%)
	5	49 (23.3%)
	6	18 (8.6%)
	7	13 (6.2%)
	8	40 (19%)

**Table 7:** The Influence of Gender on the Perception of Athletic Individuals.

Question	Option	Women Choice	Men Choice	Correlation	*p*
How do you perceive photos and videos of athletic people?	It motivates me to take action and take care of myself	50 (23.7%)	23 (10.9%)	0.22	0.004
	I feel bad because I know I look worse	61 (28.9%)	17 (8.1%)		
	I don’t pay attention to it	31 (14.7%)	29 (13.7%)		
Do you compare yourself (in terms of physical appearance) to famous, athletic people on SM?	Yes, I often compare myself	37 (17.5%)	11 (5.2%)	0.19	0.02
	It depends	70 (33.2%)	29 (13.7%)		
	I don’t compare myself to anyone	35 (16.6%)	29 (13.7%)		

**Table 8:** Impact of BMI on perception of one’s body and life.

Question	Option	BMI Underweight	BMI Normal	BMI Overweight	BMI Obesity	Correlation	*p*
Has your perception of your own body changed since you became a SM user?	Significantly changed	3 (1.4%)	41 (19.4%)	7 (3.3%)	7 (3.3%)	0.17	0.05
	Slightly changed	7 (3.3%)	58 (27.5%)	21 (10%)	9 (4.3%)		
	Did not change	5 (2.4%)	27 (12.8%)	22 (10.4%)	4 (1.9%)		
Have you ever felt that your life is less attractive than others’ while browsing SM?	Yes	2 (1%)	29 (13.7%)	11 (5.2%)	6 (2.8%)	0.07	0.9
	Sometimes	10 (4.7%)	72 (34.2%)	29 (13.7%)	12 (5.8%)		
	Never	3 (1.4%)	25 (11.8%)	10 (4.7%)	2 (1%)		

## Data Availability

Data are contained within the article.

## Abbreviations

The following abbreviations are used in this manuscript:

SM	Social media
BES	Body esteem scale
ED	Eating disorders
AN	Anorexia Nervosa
BN	Bulimia Nervosa
ON	Orthorexia Nervosa
BED	Binge eating disorder
